# Tumor-infiltrating immune cells state-implications for various breast cancer subtypes

**DOI:** 10.3389/fimmu.2025.1550003

**Published:** 2025-05-14

**Authors:** Tianshuang Xu, Hongjun Zhang, Burton B. Yang, Javeria Qadir, Hui Yuan, Ting Ye

**Affiliations:** ^1^ Department of Immunology, Mudanjiang Medical University, Mudanjiang, Heilongjiang, China; ^2^ Sunnybrook Research Institute and the Department of Laboratory Medicine and Pathobiology at the University of Toronto, Toronto, ON, Canada; ^3^ Department of Biosciences, COMSATS University Islamabad, Islamabad, Pakistan; ^4^ School of Stomatology and School of Basic Medical Sciences, Mudanjiang Medical University, Mudanjiang, Heilongjiang, China; ^5^ Department of Laboratory Medicine, The Affiliated Hospital of Southwest Medical University, Luzhou, Sichuan, China

**Keywords:** immune cell(s) infiltration, molecular subtypes, tumor microenvironment (TME), breast cancer, clinical significance

## Abstract

Breast cancer presents a variety of subtypes due to its cellular and molecular heterogeneity. The capacity of cancer cells to proliferate, invade, and metastasize depends not only on their intrinsic characters but also on their dynamic interaction with the host tumor microenvironment (TME), which includes immune cells. Meanwhile, the infiltration of immune cells in the TME severely affects the occurrence, development, treatment, and prognosis of breast cancer. Therefore, this review aims to explore the immune invasive tumor microenvironment in different intrinsic subtypes of breast cancer. Additionally, it highlights the mechanistic influence of the infiltrating immune cells on stage-wise dynamics of breast tumorigenesis. Moreover, the present review also attempts to discern the regulatory relationship between tumor infiltrating immune cells and immune microenvironment in different molecular subtypes of breast cancer, thus, spotlighting its clinical significance.

## 1 Introduction

**Figure 1:**
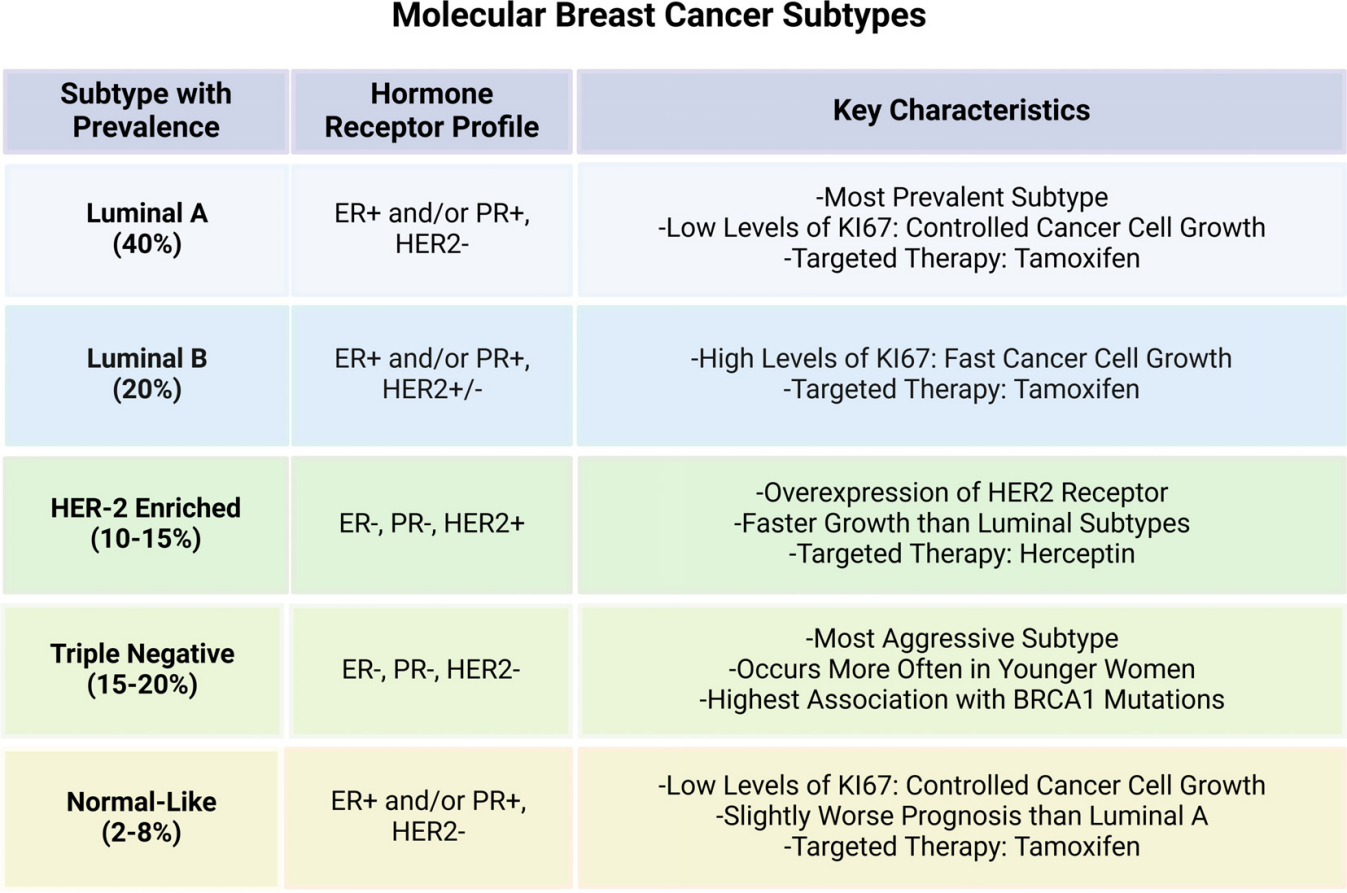
Key characteristics of the intrinsic molecular breast cancer subtypes.

Breast cancer (BC) is one of the most common malignant tumors that has posed serious threat to women’s health across the globe (1). Owing to its molecular heterogeneity, BC can be classified into distinct molecular subtypes based on the varying expression of hormone receptors i.e., estrogen receptor (ER), progesterone receptor (PR), and human epidermal growth factor receptor-2 (HER-2). The main BC subtypes include Luminal A, Luminal B, HER2 enriched and triple negative breast cancer (TNBC) and Normal-like breast cancer. Luminal A is the most prevalent subtype characterized by positive expression of ER or PR while HER-2 is negative. Luminal B subtype, on the other hand, is rather more aggressive with high proliferation index and is characterized by relatively low expression of ER/PR and increased HER-2. HER-2 enriched tumors are driven by HER2 overexpression that can be modulated through the use of targeted therapies in BC. TNBC is distinguished by the lack of ER, PR, and HER2 expression, and is the most aggressive subtype with poor prognostic value (2, 3). Normal-like has similar classical immunohistochemistry markers with luminal A tumors, but can highly express the basal epithelial genes and has worse prognosis than the Luminal A (4, 5). The molecular attributes and key features of the main BC subtypes are presented in the **Figure 1**. Hence, it is highly imperative to comprehend these subtypes for designing personalized treatment plans, thus, affecting the choice of treatment and overall patient outcome in BC.

BC progression is not only determined by the specific subtype or genomic events within the tumor cells, but also by the interaction between the cancer cell and the surrounding tumor microenvironment (TME), the stroma and various other factors (6, 7). The TME is a dynamic entity composed of both cancerous and non-cancerous cells, including fibroblasts, fat cells, endothelial cells, and immune cells (e.g. macrophages, lymphocytes, natural killer (NK) cells), soluble factors, and extracellular matrix (ECM) components (8). All the immune components which include various immune cells extracellular immune factors and cell surface molecules are specifically defined as the tumor immune microenvironment (TIME) in TME, whereby, the immune component has unique internal interactions that influence the biological behavior of tumor (9, 10), and has long been shown to be closely related to tumor development, recurrence and metastasis (11). The immune cells in the TIME establish a strong network with the BC cells, whereby, the resulting Breast Cancer Immune Microenvironment (BCIM) has a significant impact on BC development (12). A suppressive TME may potentially evade immune responses and promote angiogenesis, thus, fostering tumor progression, whereas, a pro-inflammatory TME might prompt elimination of the cancer cells (13).

Numerous research studies have shown that the differences in disease progression, chemosensitivity and patient prognosis in distinct BC subtypes are largely dependent on the interactions between the cancer cells and various components of the TME, explicitly with the tumor-infiltrating immune cells (TICs) (14, 15). For instance, poor prognosis in TNBC patients may plausibly be attributed to abundant myeloid-derived suppressor cells (MDSCs) infiltration compared to those presented with other BC subtypes (16). Similarly, increased tumor-infiltrating lymphocytes (TILs) infiltration positively correlates with increased disease-free survival (DFS) and overall survival (OS) in both TNBC and HER2-positive BC patients, however, no such correlation could be observed in Luminal A patients (17). Regardingly, high intratumoral TILs infiltration status has been demonstrated to affect the degree of infiltration for various immune cell types (18). Likewise, M2 tumor-associated macrophages (TAM2) have been shown to be a poor predictor of BC and are often associated with aggressive phenotypes in luminal and TNBC cohorts of patients (19). Therefore, it is highly significant to correctly understand and utilize the mechanistic intricacies of tumor immune infiltration in different BC subtypes.

Contextual to this, the present review aims to explore and comprehend the roles of TICs in BC, emphasizing on their functional involvement in tumor progression. Moreover, this review also highlights evolving immune-based therapeutic interventions and their impact on clinical therapeutic outcome and overall prognosis in BC patients presented with different intrinsic subtypes.

## 2 Cellular components in TME and their roles in cancer

TME is a complex ecosystem comprised of various types of cells that interact with tumor cells and influence cancer progression and therapeutic resistance. These include immune cells, cancer-associated fibroblasts (CAFs), endothelial cells, and extracellular matrix (ECM) components. Each of these cellular elements in TME undergo distinct functional changes and interact to modulate tumor progression, metastasis, and therapeutic response (20).

### 2.1 Immune cells

Under normal cellular conditions, immune cells including cytotoxic T lymphocytes (CTLs), natural killer (NK) cells, and dendritic cells (DCs) provide first line of defense against malignant transformation (21). Physiologically, these cells function in recognizing and eliminating transformed/malignant cells through antigen presentation and cytotoxic activity (22). However, tumors can potentially develop immune evasion strategies that entail (i) upregulation of immune checkpoint molecules such as PD-L1, and (ii) recruitment of immunosuppressive cells like regulatory T cells (Tregs) and tumor-associated macrophages (TAMs) (23). The functional implications of these immune cells in cancer are highly diverse. Typically, macrophages are capable of adopting an M1 (pro-inflammatory, anti-tumor) or M2 (immunosuppressive, pro-tumor) phenotype (24). In the TME, macrophages are often polarized into the M2-like phenotype, secreting anti-inflammatory cytokines such as IL-10 and TGF-β, which suppress T cell activation and promote tumor growth, angiogenesis, and metastasis (25). Tregs are recruited into the TME via chemokines like CCL22 and alleviate anti-tumor immunity by suppressing T-cell mediated responses (26). Similarly, Myeloid-Derived Suppressor Cells (MDSCs) inhibit the activation of CTLs, hence, fostering an immunosuppressive environment by secreting arginase-1, nitric oxide (NO), and reactive oxygen species (ROS) (27–29). In addition, tumor cells can put immune checkpoints such as PD-L1/PD-1 and CTLA-4 to use in order to suppress T cell-mediated cytotoxicity, thus, leading towards immune evasion (30).

### 2.2 Cancer-associated fibroblasts and ECM remodeling

Normally, fibroblasts impart critical functions in maintaining tissue homeostasis and contribute to wound healing by secreting cytokines and producing extracellular matrix proteins (31). In tumor, however, CAFs secrete pro-tumorigenic growth factors like fibroblast growth factor (FGF), TGF-β, VEGF, and IL-6, that lead to ECM remodeling, enhance angiogenesis, supporting tumor cell proliferation, invasion, and immune evasion (32). CAFs also function to create a firm fibrotic environment that potentiates metastasis (33). They also play a contributory role in mediating drug resistance by strengthening ECM stiffness and synthesizing hyaluronic acid and collagen, thus, creating a physical barrier for the drugs to penetrate (34).

### 2.3 Endothelial cells and angiogenesis

Normal endothelial cells form structured vasculature to ensure proper oxygenation and nutrient delivery to the tissues (35). Whereas in cancer, tumor-associated endothelial cells undergo abnormal angiogenesis through hypoxia-induced signaling via HIF-1α (Hypoxia-Inducible Factor-1 alpha) and VEGF secretion, hence, leading to leaky, dysfunctional, and hypoxic blood vessels, that foster tumor growth and metastasis, while impairing drug delivery and limiting its efficacy (36, 37).

### 2.4 ECM components and tumor invasion

The ECM functions to provide structural integrity and cell signaling regulation in normal tissues (38). However, in the TME, the ECM components such as collagen, laminin, and fibronectin are remodeled to advance tumor invasion (39). Enzymes like matrix metalloproteinases (MMPs) degrade the ECM, facilitating cell migration and metastasis during carcinogenesis (40). Additionally, ECM stiffness increases integrin signaling, which promotes tumor cell survival and proliferation (41). Moreover, tumor-associated ECM remodeling creates a physical barrier that restricts immune infiltration and drug penetration, hence, prompting malignancy (42).

Therefore, it is imperative to comprehensively decipher the functional implications and molecular interactions of these cellular components in the TME to provide deeper insight into tumor progression and therapeutic resistance towards immune-based strategies. More detailed research on the aforementioned entities would assist in the development of therapeutic approaches targeting the TME, such as immune checkpoint inhibitors, CAF-targeting therapies, and anti-angiogenic agents.

## 3 Immune infiltration in breast cancer

**Figure 2:**
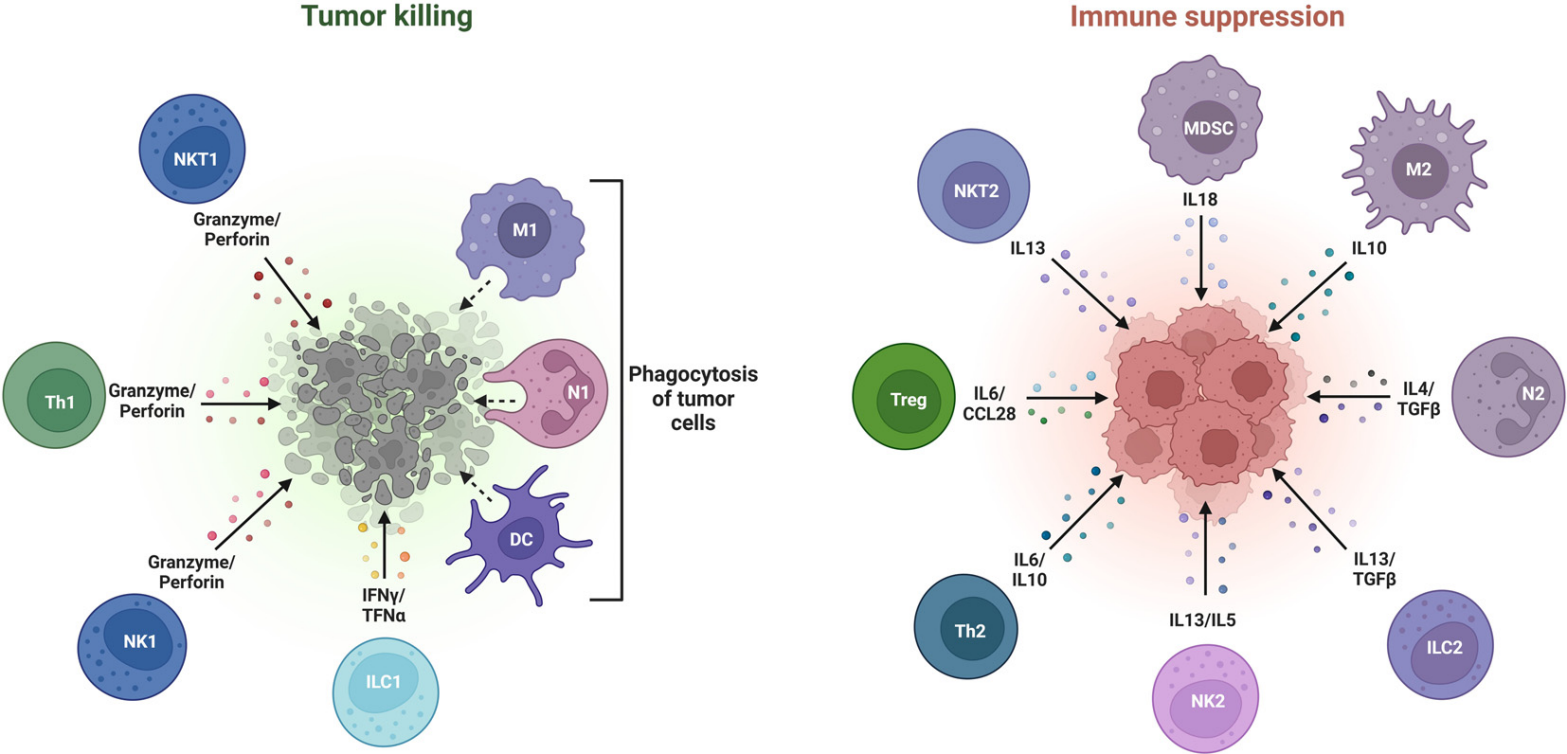
Various types of tumor-promoting and tumor-inhibiting immune cells constituting the breast tumor microenvironment.

BC progression is significantly influenced by a diverse pro-inflammatory microenvironment which is constituted of various tumor-invasive immune cells, cytokines, and growth factors. Broadly, these tumor-infiltrating immune cells (TTICs) are classified into (i) Lymphoid cells and (ii) Myeloid cells. Precisely, the lymphoid cells can promote or suppress tumor growth by modulating immune responses through B-cells, CD8+ cytotoxic T cells, CD4+ helper T-cells and regulatory T cells, while the natural killer (NK) cells employ cytotoxic activity to target cancer cells. On the other hand, the myeloid cells are comprised of macrophages with M1 (anti-tumor) and M2 (pro-tumor) phenotypes, antigen presenting dendritic cells (DCs), and myeloid-derived suppressor cells (MDSCs) that function to suppress immunity (43, 44). The equilibrium between pro-tumor and anti-tumor immune states determines the extent of cancer progression and therapeutic response in patients diagnosed with BC, thus, making TIICs potential therapeutic targets, as shown in **Figure 2**.

Accordingly, the interaction between multiple cytokines and immune infiltrating cells affects BCIM and exerts its regulatory effect to induce breast tumorigenesis and body’s protective regulatory response (45, 46). Various features of BC have long been proven to be related to the roles played by the immune infiltrating cells in BCIM. These immune cells can either exhibit anti-tumor phenotype by playing integral role in immune surveillance, or pro-tumor phenotype under the influence of TME, thus, allowing tumor escape and supporting TME to advance BC development and progression (47).

## 4 Tumor-infiltrating immune cells in intrinsic breast cancer subtypes

Significant differences have been observed in immune infiltration among different molecular subtypes of BC. Estrogens and their receptors have variable mechanisms and effects on immune cell infiltration in BC. In addition, immune infiltrating cells secrete a variety of cytokines that can affect BCIM by interacting with themselves and exert regulatory effects to induce BC or stimulate immune protection (45). For instance, BC cells can secrete IL-6 to induce the formation and maintenance of BCSCs (48), meanwhile, IL-6 induction promotes proliferation of ER positive cells, thus, presenting a more aggressive BC phenotype (49). Mechanistic studies reveal that IL-19 potently enhances BC cells proliferation *in vitro (* 50). In addition, the interaction between interleukin and immune infiltrating cells is manifested in the following aspects, i.e., the infiltration process of CD8^+^T cells can be hindered by cytokines such as IL-20 and IL-23, thus affecting inflammation and blood vessel generation in the BC microenvironment which subsequently promotes tumor progression (51). Conversely, certain interleukins exert positive effect. BC cells are often able to activate NK cells to further promote tumor substance uptake and dendritic cells (DCs) maturation, as well as their ability to produce IL-12. Moreover, IL-15 stimulation can promote the activation of NK cells and the maturation of DCs in BCIM, thus, combined administration of IL-15 can enhance the therapeutic effect of drugs on BC patients (52).

**Figure 3:**
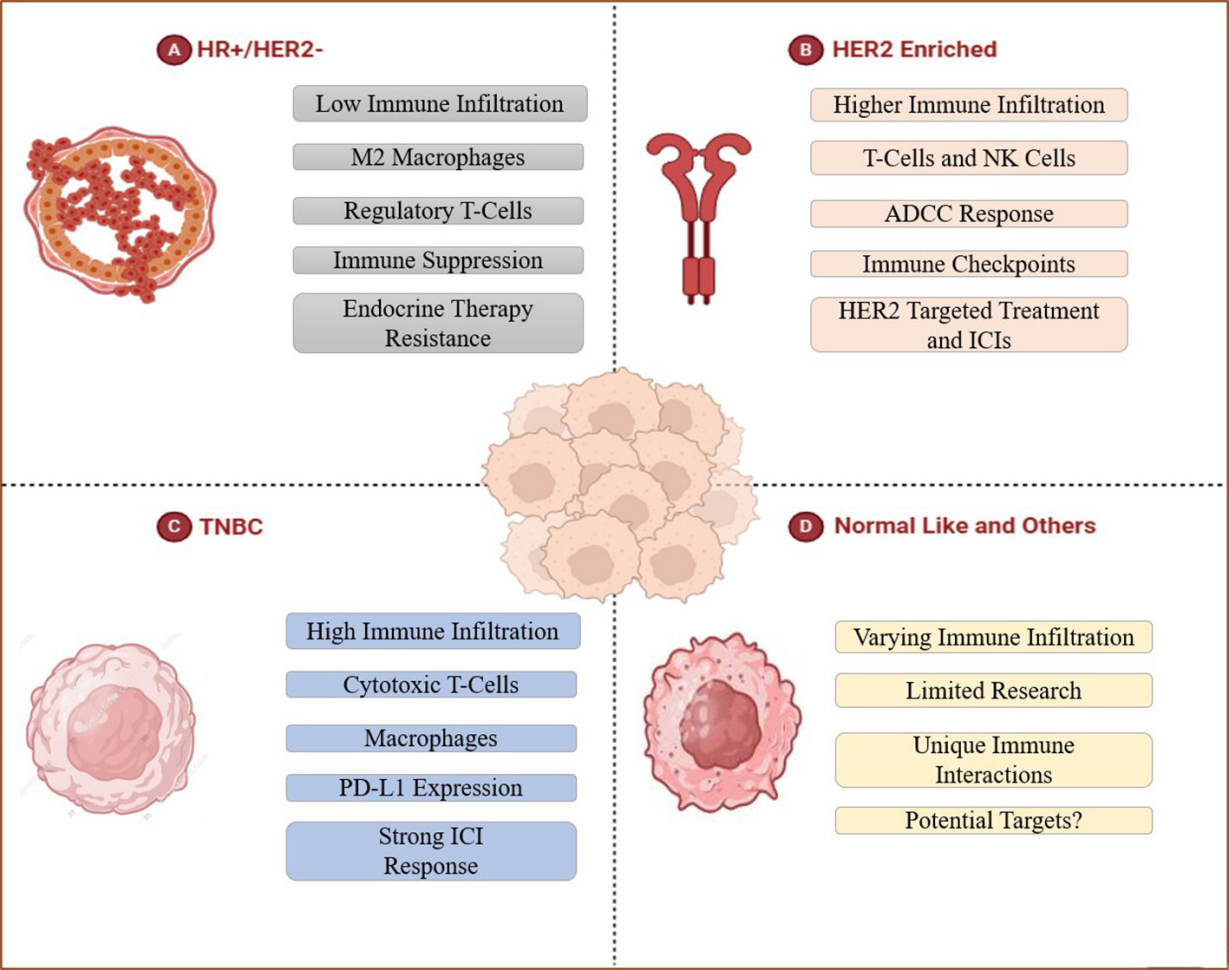
Tumor-infiltration in distinct intrinsic breast cancer subtypes. **(A)** HR+/HER2- BC represents endocrine therapy resistance with low overall TIICs that are mainly rich in M2 macrophages and Tregs, which inhibit anti-tumor immune response and mediate immune evasion. **(B)** The higher immune infiltration of HER2 enriched BC includes T-cells and NK cells, this subtype shows ADCC response, can be treated in the targeted way. **(C)** TNBC contains large amounts of TILs (specifically macrophages and cytotoxic T cells) and expresses PD-L1, thus can be treated with immune checkpoint inhibitors. **(D)** Researches about Normal-like and other subtypes of BC are limited, this part remains to be discussed.

Distinct molecular subtypes of BC may contain varying proportions of the tumor-infiltrating immune cells (TIICs), thereby, regulating the degree of disease aggressive and prognosis, as described in the following sub-sections. (**Figure 3**, **Table 1**).

**Table 1:** The distinction of immune infiltration in different BC subtypes.

Subtype of BC	Highly infiltrated immune cells	Lowly infiltrated immune cells	References
ER^+^ BC	NK, Neutrophil	CD8^+^T, CD4^+^T	(19)
ER^-^ BC	Tregs, TAM2, MCs	CD8^+^T, CD4^+^T B lymphocytes, DCs	(55, 56)
TNBC	TILs, MDSCs	—	(16, 58, 62, 63)
HER2^+^ BC	DCs, MCs, Tregs, Neutrophil	—	(57, 58)

ER^+^BC, ER-positive breast cancer; ER^-^BC, ER-negative breast cancer; TNBC, triple-negative breast cancer; HER2^+^BC, HER2-positive breast cancer; NK, natural killer cells; CD8^+^T, cytotoxic T cells; CD4^+^T, memory T cells; Tregs, regulatory cells; TAM2, M2 tumor-associated macrophages; MCs, mast cells; DCs, dendritic cells; TILs, tumor-infiltrating lymphocytes; MDSCs, Myeloid-derived suppressor cells.

### 4.1 TIICs in hormone receptor-positive (HR+/HER2-) breast cancer

HR+/HER2- BC patients are typically less responsive towards immunotherapy owing to low degree of TIICs. TME in such BC subtype is predominantly rich in M2 macrophages and Tregs that promote tumor growth by repressing anti-tumor immune response and mediating immune evasion (53). Moreover, such patients show increased resistance towards endocrine therapy like aromatase inhibitors and/or tamoxifen due to presence of macrophage-induced inflammation, thus, restraining their therapeutic efficacy (54). In similar regards, ER-positive BC patients present higher proportion of NK cells and neutrophils than other tumor infiltrating immune cells such as cytotoxic T cells (CD8^+^T) and memory T cells (CD4^+^T) (55). Comparably, ER-negative BC patients are primarily richer in regulatory T cells (Tregs), TAM2, and activated mast cells, while the cells associated with better prognosis such as CD8^+^T, CD4^+^T, B lymphocytes, and DCs are less abundant (56). Hence, the therapeutic outcomes in BC patients exhibiting luminal phenotype can be improved by targeting immunosuppressive cells and enhancing T cell activation, providing novel insights for combinatorial therapies with endocrine agents and immune-based therapies (**Figure 3A**).

### 4.2 TIICs in HER2-enriched breast cancer

HER2-enriched BC often presented with high degree of immune infiltration in comparison with the luminal BC subtypes. Such tumors exhibit significantly increased proportion of NK cells and T-cells (57). Studies have reported that DCs, mast cells (MCs), Tregs and neutrophils are associated with poor prognosis, disease recurrence and metastasis (58). However, there are not too many reports on HER2-positive BC-related immune infiltrating masses. Strong infiltration of immune cells in HER-2 amplified breast tumors augments the response to HER2-targeted agents such as trastuzumab and pertuzumab, thus, prompting tumor cell death by means of antibody-dependent cellular cytotoxicity mechanism (59). However, the upregulation of immunosuppressive pathways may culminate in developing resistance, which can be overcome by the simultaneous use of ICIs like anti-PD-1/PD-L1 antibodies with HER-2 targeting agents, thus, improving patient outcomes by reinforcing the anti-tumor immune response and increasing therapeutic efficacy (60) (**Figure 3B**).

### 4.3 TIICs in triple negative breast cancer

TNBC, being an aggressive subtype of BC, lacks expression of the hormone receptors and ERBB2 (61). TNBC is characterized by increased immune infiltration and contains more TILs (particularly macrophages and cytotoxic T-cells) than other breast cancer subtypes (58) and exhibits a highly infiltrating state of MDSCs (62). Extensive research has reported a strong correlation between high TIICs proportion and augmented response to immunotherapy in TNBC, thus, endorsing TNBC as the most suitable subtype for immune-based therapies (63). Such BC tumors can easily evade attacks by the immune system by expressing Programmed death-ligand 1 (PD-L1) (64), whereby, using immune checkpoint inhibitors (ICIs) in combination with chemotherapy or other immune modulators can potentially enhance treatment efficacy by restoring T-cell function, thus, providing a therapeutically effective intervention for TNBC management (65) (**Figure 3C**).

### 4.4 TIICs in normal-like and other breast cancer subtypes

Normal-like and other subtypes of BC(e.g. HER2-low BC, HER2-zero BC) demonstrate varying degrees of immune infiltration, making it challenging to anticipate immune responses in such patients. The molecular features like resemblance with normal breast tissue, downregulation of luminal genes, low KI67 index, decreased TP53 mutations, and restricted growth factor signaling activation, may plausibly lead to restricted response to immunotherapy (66, 67). Moreover, the interactive interplay between the highly invasive immune cells and BC progression in normal-like subtypes is still elusive, demanding more extensive research in future. Therefore, it is highly crucial to identify explicit immune markers and discover putative targets for advanced treatment approaches and improved patient survival (**Figure 3D**).

## 5 Impact of variations in immune infiltration on intrinsic BC subtypes

### 5.1 Immune infiltration in TIME and pathogenesis of intrinsic BC subtypes

Immune infiltrating cells in tumors can regulate the pre-tumor inflammatory microenvironment by synthesizing and secreting some cytokines, participate in affecting tumor-related signaling pathways or blocking tumor immune response, and create conditions for tumor formation (68). The immune microenvironment in breast cancer is heterogeneous and in a dynamic state, and the differences can be observed in the molecular subtypes of breast cancer and the disease environment (69).

**Figure 4:**
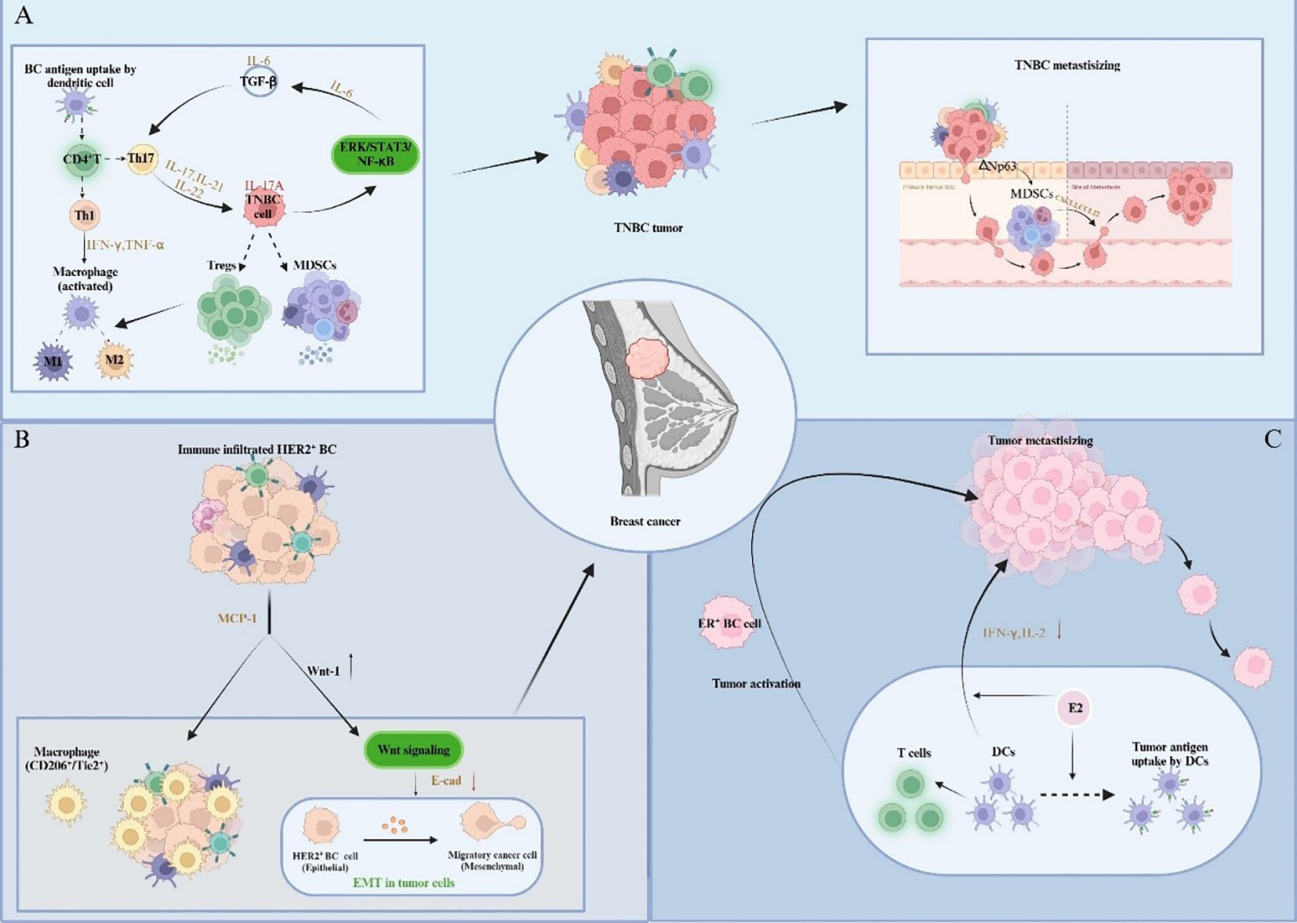
Impact of immune infiltration on the pathogenesis and development of different BC subtypes. **(A)** Cytokines participate in intercellular signaling, mobilize and recruit immune cells to infiltrate, thereby promoting inflammation and forming immunosuppressive TIME, and further promoting the metastasis of TNBC. **(B)** Tumor cells and immune cells trigger precancerous lesions of HER2-positive breast cancer through cellular communication and signal transduction. **(C)** Estrogen regulates the immune ability of immune cells and triggers the activation and migration of ER-positive breast cancer.

During the early stage of TNBC progression, the patient’s body is stimulated by damage, which leads to preliminary local inflammatory response, and the activated DCs in BCIM can transfer antigens to local lymph nodes (armpit, inner milk) to promote adaptive CD4^+^T cell activation to Th1 and Th17 (70). Th1 cells can secrete Interferon-γ (IFN-γ) and tumor necrosis factor α (TNF-α) to activate pro-inflammatory M1 macrophages (71), while Th-17 cells produce cytokines IL-17, IL-21, and IL-22. The inflammatory cytokine IL-17A binds to TNBC cells and activates the ERK, NF-κB and STAT3 pathways with carcinogenic properties, ultimately leading to IL-6 production. Combined with TGFβ, IL-6 can further activate Th17 cells, induce chronic inflammatory states and enhance IL-17A signaling effects (72). Additionally, TNBC cells can recruit MDSCs to aggregate, whereby these MDSCs promote TNBC growth by promoting immunosuppression, angiogenesis, and inflammation. Comparably, MDSCs attract Tregs and promote the production of TAM2, thus, resulting in a strong immunosuppressive TME, which provides a favorable environment for TNBC (73) (**Figure 4A**).

With chronic inflammatory microenvironments, ER-negative and TNBC cells can prevent the immune function of a variety of immune infiltrating cells, and also recruit a variety of immunosuppressive cells (such as MDSCs, M2, etc.). By utilizing the cytokines (such as IL-17A), chemokines and inflammatory mediators produced by these immunosuppressive cells, a microenvironment rich in pro-angiogenic factors and resistant to immune responses suitable for tumorigenesis is created (74, 75). In addition, studies in HER2-positive BC mouse models have uncovered a novel premalignant mechanism, that is, in precancerous lesions, monocyte chemotactic protein-1 (CCL2) produced by cancer cells and myeloid cells attracts CD206^+^/Tie2^+^ macrophages and induces the upregulation of WNT-1 to stimulate Wnt signaling. Subsequently, E-cadherin ligations in HER2-positive early BC cells are then down-regulated, resulting in EMT-like responses that favor tumorigenesis (76) (**Figure 4B**).

### 5.2 Immune infiltration in TIME and development of intrinsic BC subtypes

There are differences in the specific immune infiltration status of different types of BC, which may have variable effects on tumor growth, invasion and metastasis. In the early stage of BC development, the body’s immune system can still produce an adequate immune response against tumor antigens. However, the specific reaction of different BC subtypes is distinctive. In ER-positive BC, as a key hormone involved in the progression of BC, estrogen can upregulate the expression and secretion of various inflammatory cytokines and chemokines, enhancing the antigen presentation ability of DCs (77). While DCs can also secrete pro-inflammatory cytokines to stimulate T lymphocytes, hence triggering the tumor activation and migration processes (78). Meanwhile, the effect of E2 on DCs depends on its mature stage. Hormone exposure reduces interferon-gamma and interleukin-2 produced by mature DCs, thereby accelerating breast tumor progression (79) (**Figure 4C** **).**

Since TNBC cells often express elevated levels of ΔNp63(a transcription factor of the p63 gene with the ΔN isomer (activation domain)), the number of MDSCs are increased in TNBC patients compared to other molecular subtypes of BC. This may be attributed to enrichment of MDSCs in TNBC patients under the drive of transcription factor ΔNp63 (80). As a heterogeneous population of progenitor and progenitor cells of bone marrow cells, MDSCs can help tumors form a premetastatic microenvironment and promote tumor metastasis by promoting angiogenesis and tumor cell invasion (81). In addition to this, MDSCs can also promote the metastasis of TNBC cells by activating chemokine CXC ligand 2 (CXCL2) and macrophage derived chemokine (CCL22) directly (82). In the meantime, Tregs can work with MDSCs to counter tumor immune response and promote tumor development through immunosuppressive crosstalk induced by Mast Cells (MCs) in TNBC (83). These cells can disrupt the host’s immune response through multiple mechanisms involving cell-to-cell contact and the production of immunosuppressive cytokines and metabolites, thereby sustaining tumor progression and aggressiveness (84). The specific infiltration state of such cells can also affect the classification and staging of TNBC. TNBC is characterized by high mutation rate and high expression of programmed cell death - ligand 1 (PD-1). Contextual to this some research studies have demonstrated that the expression of PD-L1 in TNBC tissues is correlated with both CD4^+^TILs and CD8^+^TILs using immunohistochemical staining and immunofluorescence double staining techniques. PD-L1-CD8^+^TILs infiltration is an independent predictive factor affecting OS in TNBC patients, whereby this type of TILs infiltration predicts smaller tumor diameter, low histological grade, low TNM stage, no lymphatic vessel invasion and no lymph node metastasis, and better prognostic outcome.

In basal-like breast cancer, the expression of chemokine CXCL12 and its receptor CXCR4 is increased significantly under hypoxic environment compared to other BC subtypes, which increases the degree of infiltration of CD4^+^T cell subsets with significant immunosuppressive effect, such as Tregs. This is associated with the malignant progression of basal-like breast cancer (85). Besides, it has been suggested that in breast cancer cases involving BRCA1 mutations, excessive lymphocyte penetration only exaggerates phenotypic features that are not associated with disease progression (86). Therefore, it is necessary to further elucidate the mechanism of lymphocyte aggregation within BC for exploring the response and prognosis in BC patients presented with mutations.

## 6 Clinical implications of TIICs in devising subtype-specific interventions for BC management

### 6.1 Therapeutic targeting of TIICs in intrinsic BC subtypes

The immune infiltration characteristics of BC affect treatment outcome in patients by altering the holistic process of tumor occurrence and development. More and more targeted treatment strategies are designed according to the patient’s immune infiltration mechanism and characteristics, which have achieved enhanced therapeutic outcomes in BC patients (87). Chemotherapy and radiotherapy are often the first-line of treatment options once the patient is diagnosed with BC. Considering the process of interaction between inflammatory factors and various immune infiltrating components in BC (88, 89), the expression of NLRP3 inflammasome and inflammatory factors in BC epithelial cells and innate immune cells and so on (90), it is highly preferred to select inhibitors that directly target the NLRP3 protein to boost the effectiveness of chemotherapy and radiotherapy by modulating the immune system (91). In addition, a variety of therapeutic approaches for different immune infiltrating characteristics in various BC subtypes are becoming increasingly mature, and have achieved significant curative effects (92).

Among various subtypes of BC, high expression of programmed cell death ligand 1 (PD-L1) in TNBC is one of the key research hotspots in BC immunotherapy (93, 94). The PD-L1 combination therapy which includes immune checkpoint inhibitors (ICIs) and the antibodies of anti-programmed cell death protein 1 (PD-1) for TNBC are often used to enhance the effectiveness of CAR immune cells (95). Additionally, HER2 overexpression induces pro-inflammatory signaling, fostering an immune-permissive TIME characterized by elevated PD-L1 and TILs expression in responsive patients (96, 97). Therefore, combined blocking of HER2/immune checkpoint can be selected for HER2-enrich BC. Clinical studies have confirmed that trastuzumab combined with PD-1 inhibitors can enhance the therapeutic effect of HER2-positive patients, because trastuzumab can activate the innate immunity of her2+ patients through antibody-dependent cellular cytotoxicity (ADCC) (98). In contrast, low immune infiltration of luminal BC and low expression of TILs and PD-L1 (99) lead to patients being insensitive to immunotherapy, but the therapeutic effect can be enhanced by adjusting the microenvironment by increasing antigen presentation (100) and reversing immunosuppression (101).

**Figure 5:**
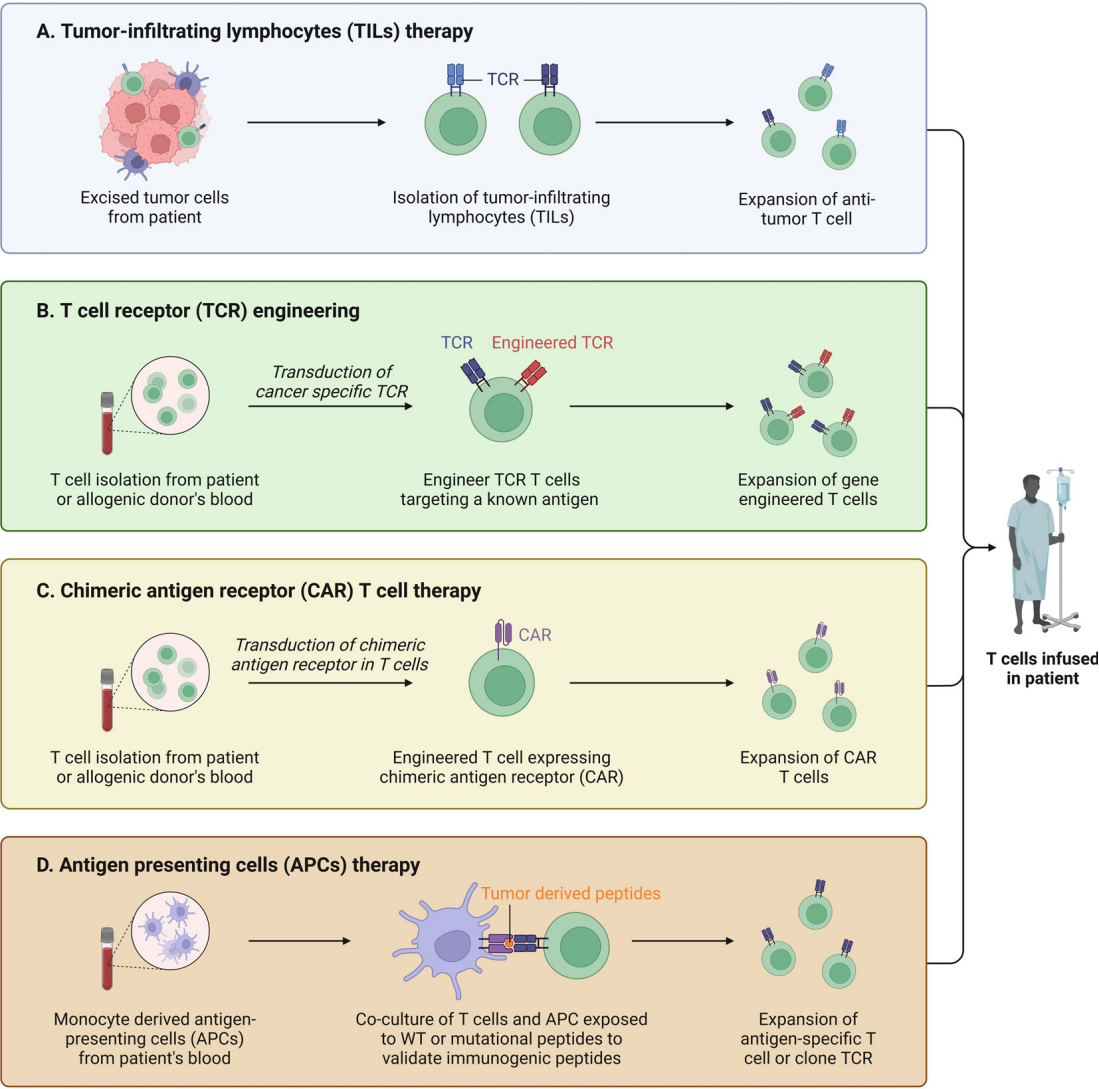
T cell based immunotherapy. **(A)** TILs were isolated from tumor tissue and used for treatments with enhanced immunity after induction. **(B)** T cells are isolated and collected and genetically engineered to target cancer cells. **(C)** Isolated autologous or allogeneic immune effector T cells, activated and expanded *in vitro*. **(D)** Artificial antigen-presenting cells were constructed and activated for tumor therapy.

Besides, the therapeutic approach targeting T cells in BC has brought good news to the majority of BC patients. CAR is a synthetic cell surface receptor that helps immune cells recognize tumor cells (102). Moreover, it can help modify T cells to recognize tumor-specific antigens and eventually dissolve tumor cells when introduced into T cells by special techniques (103). Zhao et al. (104)found that the co-expression of constitutionally activated interleukin-7 receptor (C7R) can significantly improve the activation, cell proliferation and cytotoxicity of CAR-T cells through anti-tumor experiments in mice. They also proved that the enhanced CAR T cells showed significant antitumor activity in the subcutaneous xenotransplantation model of TNBC through *in vivo* experiments, providing a new strategy for the treatment of patients diagnosed with TNBC (**Figure 5**). The human epidermal growth factor receptor 2 (HER2; also known as HER-2/neu or ErbB2) is a member of the transmembrane epidermal growth factor receptor family and is one of the most studied tumor-related antigens in tumor immunotherapy. In order to address the issue of insufficient efficacy of monoclonal antibodies targeting HER2 in BC patients, Sun et al. (105) successfully designed a novel humanized HER2-CAR-T cell using a multi-step overlapping extension polymerase chain reaction scheme and demonstrated its inhibitory effect on HER2 positive tumor cells *in vitro* and induced experimental BC regression *in vivo*. Nonetheless, there is still an enormous room for development and exploration value in the field of studying clinical treatment methods for patients based on the characteristics of immune infiltration in different subtypes of BC.

### 6.2 Prognostic significance of TIICs in intrinsic BC subtypes

The infiltration characteristics of immune cells in BC can not only provide new strategies for clinical treatment of patients, but also affect the prognosis of BC patients. For example, in addition to providing treatment for BC patients, the high infiltration of TILs has been confirmed to be positively correlated with good prognosis and high pathological complete response (pCR) in patients with different BC subtypes in a large number of studies reported in the recent years (106). Although HER2-positive BC exhibits relatively low TILs density, its HER2-driven pro-inflammatory signaling actively recruits macrophages and T cells to reshape the tumor microenvironment (107). Notably, anti-HER2 therapies dynamically upregulate both TILs infiltration and PD-L1 expression, thereby reversing immune suppression, significantly enhancing pCR and prolonging patients’ survival, as demonstrated in large-scale clinical trials (108, 109). TNBC demonstrates significantly heightened immunogenicity compared to ER-positive/HER2-negative BC, owing to its enriched tumor microenvironment characterized by elevated TILs, pro-inflammatory mediators (e.g. IL-6, CXCL10), and CD8^+^T cells (18). Clinical investigations have substantiated that increasing TILs density enhances chemotherapy sensitivity and activates anti-tumor immune response, thereby reducing mortality risk and disease recurrence in TNBC patients (110). Reportedly, TNBC has more TILs than ER positive/HER2-negative breast cancer (111). Moreover, TNBC patients with high CD8^+^T cell infiltration tended to show higher survival rates, and this correlation is absent in other BC subtypes, therefore, CD8^+^T cell score can be used as a prognostic biomarker for TNBC management (112). On the contrary, regulatory T cells account for about 5-10% of CD4^+^T cells, and can suppress the proliferation of CD8^+^T cells and the production of cytokines (113). The increase in TILs is positively correlated with Disease free survival (DFS) and overall survival (OS) among TNBC and HER2 positive BC patients who had received neoadjuvant chemotherapy; however, this correlation is not established in Luminal A type BC. This may be ascribed to the impact of endocrine therapy on the immune system of the BC patients exhibiting Luminal A subtype (114). Therefore, the evaluating the interplay between the number of CD8^+^T cells and regulatory T cells is likely to be a predictive and prognostic factor for TNBC.

Besides TILs, NK cell infiltration status in BCIM can also be used to assess patient prognosis, as they are involved in innate immunity and can recognize and kill altered cells without prior sensitization. NK cells can recognize and eliminate cells that do not express MHC Class I, so that BC cells could escape from the cytotoxic effects mediated by T cells in this mechanism (115). The balance between the pro-tumor and anti-tumor activities of NK cells varies among different subtypes of BC due to various reasons. A higher proportion of NK cells and neutrophils was observed in ER positive breast tumors, while the proportion of immune infiltrating cells associated with good prognosis, such as cytotoxic T cells (CD8^+^T) and immature and memory T cells (CD4^+^T), is relatively small (116). Therefore, strong infiltration of NK cells in ER positive and HER2 positive BC patients is often associated with a good prognosis. Contrastingly, this strong infiltration phenomenon often indicates an unfavorable prognosis in TNBC patients. In ER-negative BC, the dominant immune infiltrates predominantly comprise Tregs, TAMs and activated mast cells-immunosuppressive populations consistently linked to adverse clinical outcomes (117).

It is worth noting that BC has long been considered “ immunologically quiescent “ or “cold tumor” because the TILs density in each subtype of BC is generally lower than that in other types of tumors, thus resulting in a decline in the body’s immune response (118). However, this view is not comprehensive, because BC can vary between different patients, different subtypes, and different disease settings (early breast cancer and metastatic breast cancer). Alexandra Thomas et al. (119) demonstrated that elevated TILs density in highly proliferative Luminal B, HER2-enriched and basal-like subtypes correlates with enhanced anti-tumor immunity, showing strong prognostic value in some patients. Regardless of the BC subtype, highly infiltrated tumors with TILs can always induce an efficient immune response (114). The robust infiltration of TILs in TNBC has been established as a pivotal prognostic biomarker, with higher TILs density strongly correlating with improved survival outcomes and therapeutic responsiveness in TNBC patients (120). However, only TILs can significantly predict tumor pCR in ER-positive/HER2-negative breast cancer, which is different from TNBC (121). Research studies employing transcriptomic analysis have demonstrated that TILs are positively associated with an enhanced anti-tumor immunity in ER-positive/HER2-negative breast cancer subtypes, as well as with better overall survival in HER2-positive and TNBC subtypes (122). HER2-positive BC exhibits a distinct immunosuppressive phenotype characterized by hormone signaling-mediated suppression of antigen presentation, enhanced infiltrating of immunosuppressive cells, and depleted TILs population, thus, traditional immune indicators such as TILs have limited value in predicting its survival (96, 97). Nevertheless, emerging evidence demonstrates that it can improve the prognosis of HER2-positive BC patients by activating the immune microenvironment with targeted drugs (98). In summary, the specific prognosis of patients with different BC subtypes may be different due to the immune cell infiltration of the body, thereby exerting differential impacts on clinical research.

## 7 Limitations and future prospects

Despite a putative role of TIICs in the prognostic and therapeutic management of BC, several challenges still persist in regards to translating immune-based therapies into a clinical success. One of the major constraints in the development of mechanisms for immune evasion, whereby, the tumor cells plausibly inhibit immune responses by overexpressing immune checkpoints such as PD-L1 and CTLA-4, therefore, engaging Tregs and fostering an immunosuppressive TME (123). Thereupon, it is highly imperative to overcome such constraints through the use of combinatorial therapies for prompting immune activation in BC, which include the use of ICIs in conjunction with chemotherapeutic agents (124), HER-2 targeted antibodies, and/or personalized cancer vaccines (59).

Given the heterogenous nature of BC, it is crucial to develop tailored immunotherapies. On one hand, TNBC responds well to immune checkpoint blockade while the luminal breast tumors often have low degree of immune infiltration (125). This necessitates the need to augment T-cell recruitment in these cells and strategizing therapeutic efficacy in HR+/HER2- BC. Likewise, HER2-enriched tumors may exhibit increased immune activity, but may also develop therapeutic resistance (126), highlighting the requirement for novel adaptive immune interventions. Pertinently, the success of immunotherapy can be maximized by screening biomarkers for patient selection across various BC subtypes (127).

In-depth insight into the composition and functional implications of TIICs has been made attainable due to recent advances in breakthrough technologies such as single-cell analysis and spatial transcriptomics (128, 129). Such technological interventions facilitate the process of precise immune profiling, thus, making it possible to comprehend pro-tumor and anti-tumor immune states and devising therapeutic measures (130). Forthcoming research studies can explore new immune targets and prompt next-generation immune-based treatments specifically customized with respect to intrinsic BC subtypes by integrating such approaches (131). These interventions will be helpful in overcoming the challenges encountered in regards to therapeutic efficacy, durability, and improved patient outcomes, thus, guiding a way to precision immuno-oncology in BC care.

## 8 Conclusion

Breast cancer progression is largely determined by the changes in its genome and the tumor immune microenvironment. The dynamic changes in immune cells and the immune components of BC always run through the whole process of breast cancer development, which plays a role in hindering or promoting tumors, and can seriously affect the treatment and prognosis of the patients. The immune infiltration status of breast cancer is different in various breast cancer subtypes, which affects the pathogenesis, disease progression and therapeutic outcome in each type of breast cancer, nevertheless, the reasons and mechanisms underlying these variable responses still remain to be explored.

In conclusion, tumor-infiltrating immune cells (TIICs) impart critical functions in BC progression, therapeutic response and overall prognosis with different immune landscapes across distinct intrinsic BC subtypes. Specifically, TNBC demonstrates strong immune infiltration and response towards immunotherapy, whereas, breast tumors exhibiting luminal phenotype remain immunologically cold. Pertinently, advancement in the use of ICIs, CAR-T cell therapy, and TIIC profiling also presents therapeutic promise in furnishing subtype-specific interventions for BC management. In the recent years, targeted treatment methods based on the immune infiltration characteristics in patients with different BC subtypes have made new breakthroughs and achieved promising results, however, inadequate treatment, poor prognosis and recurrence are some of the problems that are still required to be addressed and resolved. Overall, the discussion and analysis of immune cell infiltration in different subtypes of breast cancer is helpful to decipher immune cell plasticity across BC progression stages, identify dominant immune checkpoints within subtype-specific ecosystems, develop biomarker-driven combination regimens, so that we can better understand the development of the disease and formulate appropriate treatment strategies for BC management, which has important research significance. Nonetheless, it is highly necessitated to focus on unveiling molecular mechanisms for disabling immune evasion, precision immunotherapies, and exploiting cutting-edge technologies in future research.
